# Treating Visual Inattention in Acute Stroke Survivors Using a Therapy Scanning Wall: A Proof-of-Concept Study

**DOI:** 10.22599/bioj.311

**Published:** 2023-08-09

**Authors:** Natalie Sharp, Lauren R. Hepworth

**Affiliations:** 1 Leighton Hospital, Mid Cheshire Hospitals NHS Foundation Trust, Crewe, UK; 2 Institute of Population Health, University of Liverpool, Liverpool, UK

**Keywords:** visual inattention, stroke, rehabilitation, screening, visuospatial neglect

## Abstract

**Background:**

Visual inattention is common following right hemisphere stroke, with up to 80% of patients being affected. Visual inattention following stroke is linked to poorer outcomes. There is no clear evidence for how visual inattention should be treated in the hospital inpatient setting.

**Objective:**

To explore the practical implications and possible benefits of using a visual scanning wall in a stroke rehabilitation unit as an assessment and treatment tool for visual inattention.

**Methods:**

This proof-of-concept study recruited stroke survivors with visual inattention. Participants used the scanning wall for scanning training five days a week for two weeks. Assessments using the scanning wall and modified Albert’s test were conducted at baseline and at day 14. Both participants and staff delivering the training were asked to complete an acceptability questionnaire.

**Results:**

All participants demonstrated an improvement in the number of pictures identified from baseline to day 14. There was a mean improvement of 9.20 (95% CI 4.77 to 13.63) in the 14 days. This is a statistically significant improvement in the scanning wall score between baseline line and day 14 (*p* = 0.01). All participants and staff reported the scanning wall as acceptable to use.

**Conclusion:**

This proof-of-concept study has demonstrated the scanning wall could be used to assess for visual inattention in extra personal space. Also, it could be beneficial and is acceptable for the treatment of visual inattention within a hospital inpatient setting for acute stroke survivors.

## Background

Currently more than 100,000 strokes occur a year in the UK. Two thirds of stroke survivors leave hospital with a disability, totalling an estimated cost of 26 billion pounds a year (Stroke Association 2022).

Visual inattention is an impairment in which individuals do not attend to visual stimuli or do not explore the visual half-space contralateral to the cerebral lesion. It can manifest in the individual peri personal (i.e. reaching distance) or extra personal (i.e. beyond reaching distance to far distance) (Halligan et al. 2003; Ten Brink et al. 2019; Van der Stoep et al. 2013). Visual inattention is linked to poorer outcomes for stroke patients and a large cause of disability (Fasotti & van Kessel 2013). Visual inattention is common in acute stroke survivors, with up to 82% of stroke survivors being affected (Hepworth et al. 2016). Visual inattention has been linked with increased risk of falls, longer length of stay and a reduced likelihood of returning home (Chen et al. 2015). An individual’s functional ability and independence is impacted by visual inattention and their ability to interact within their surrounding environment (Maxton et al. 2013). It is therefore critical that the presence of visual inattention is detected to allow timely therapy (Moore et al. 2022). The National Clinical Guidelines for Stroke recommend that visual inattention should be treated. However, current evidence does not support a particular rehabilitation method (Intercollegiate Stroke Working Party 2016; National Institute for Health and Clinical Excellence 2013). A recent Cochrane review assessed a wide variety of intervention options for spatial inattention, including visual inattention, but due to lack of high quality evidence the effectiveness of these remains unproven (Longley et al. 2021).

An international survey of clinical practice with regard to screening for spatial inattention analysed the responses of 454 stroke care professionals (Checketts et al. 2021). The most popular types of assessment were cognitive tasks such as line bisection and cancellations tasks, followed by functional assessment with a wide variety of specific assessment tasks included under these two categories (Checketts et al. 2021).

The limitation of using pen and paper cognitive screening tasks is that they are primarily assessing in an individual’s immediate peri personal space and do not address visual inattention in the wider environment, or in an individual’s extra personal space (Azouvi 2017; Butler et al. 2009). The European Academy of Neurology recommends the use of multiple pen and paper tasks in addition to a functional assessment, time permitting (Moore et al. 2022). The scanning wall was developed to provide a standardised screening assessment for extra personal visual inattention and fill the gap between pen and paper tasks and functional assessment.

Therefore, the aim of this study was to explore the practical implications and possible benefits of using a visual scanning wall in a stroke rehabilitation unit as an assessment and treatment tool for visual inattention.

## Methods

This was a prospective observational proof-of-concept study to explore the practical implications and possible benefits of using a therapy scanning wall. This was a new assessment and treatment innovated for use with stroke survivors with visual inattention whilst an inpatient.

Stroke survivors were recruited from a stroke unit at a district general hospital comprising eight acute and 20 rehabilitation beds.

**Figure 1:**
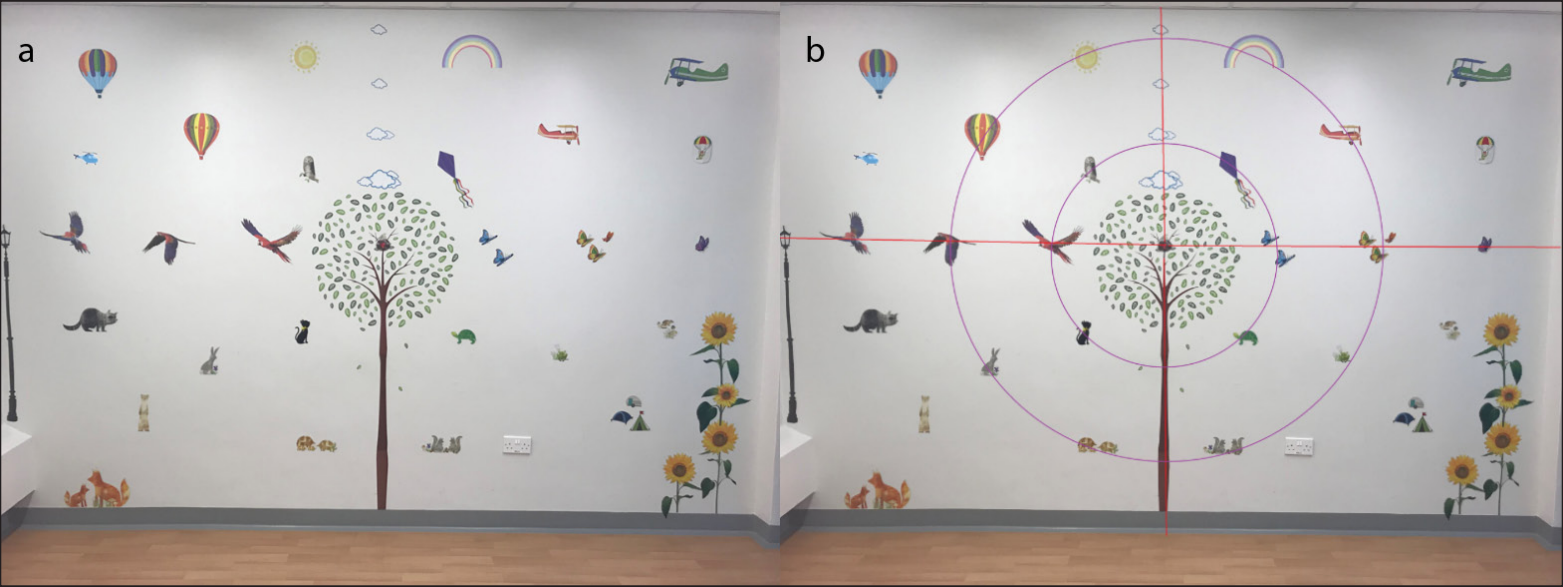
Picture of the therapy scanning wall as seen in person (a) and with outlay of positioning (b).

The therapy scanning wall was set-up in a quiet environment, minimising the influence of auditory stimulus. Wall stickers were used to create the wall. Pictures were positioned on a 2.7 m × 4 m wall (Figure 1). The centre point of the wall was marked with a small, red picture. The wall was divided into quadrants, representing the quadrants of the visual field. Each quadrant had the same number of pictures in each. The wall was further divided into three concentric circles with the same number of pictures in each. These were carefully measured to ensure a standard distance from the centre of the circle. Participants were positioned at a distance of 2.5 metres from the wall in line with the centre. The wall therefore spanned 77.3° of the visual field horizontally and 61.6° vertically.

Adults (≥18 years old) admitted with an acute clinical or radiological diagnosis of stroke and identified to have visual inattention during therapy assessment, with an expectation of an inpatient stay of at least two weeks, were considered for inclusion. Stroke survivors who were medically unfit, had previous deficits in vision, cognitive deficits or expressive/receptive language difficulties which impacted on their ability to participate, or did not speak/read English adequately, were excluded. Ability to participate was determined from the occupational therapy and speech and language therapy assessments.

This study was undertaken in accordance with the Declaration of Helsinki with UK NHS research ethics approval (REC 20/YH/0075). Written informed consent was obtained prior to participation. All stroke survivors received standard care, with study participants receiving additional assessment and therapy using the scanning wall.

Demographic information including age, sex, type of stroke, laterality of stroke and National Institute of Health Stroke Scale (NIHSS) on admission were recorded. The NIHSS score is widely used to demonstrate severity of stroke, ranging from 0–42 with a higher number indicating a greater stroke severity. At baseline, participants completed the modified Albert’s test, which is commonly used at the bedside to assess visual inattention (Zeltzer & Menon 2010). The maximum score for the modified Albert’s test is 40 and the maximum score for the scanning wall is 30. A higher number indicated the identification of more lines or pictures, and therefore less effected by visual inattention.

**Figure 2:**
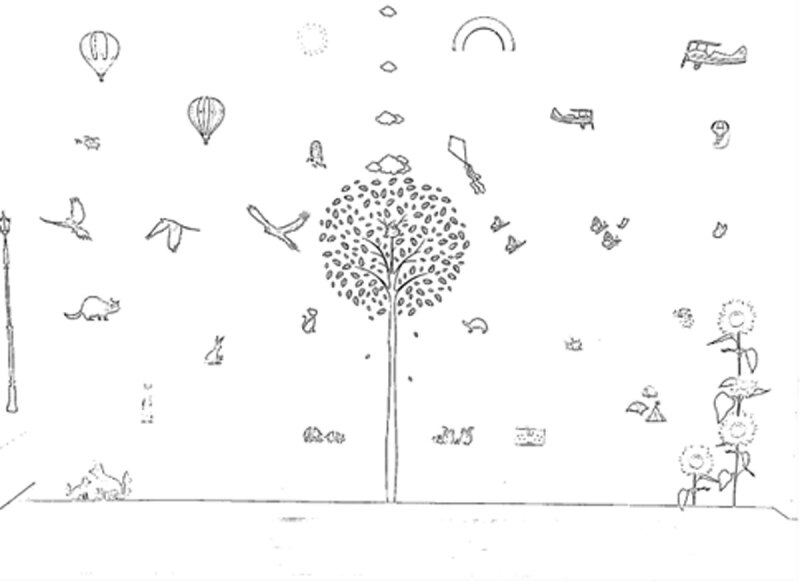
Example of paper recording sheet for the scanning wall.

During the baseline, assessment participants were read a brief instruction before entering into the room where the scanning wall was situated. A chair for the participant was set at 2.5 m away, directly facing the middle of the wall. The therapist sat directly behind the participant, so not to influence scanning from visual or auditory cues. The participant was asked to name all of the individual pictures they could see. Participants were assessed using the wall with a standard script, so all participants received standardised information and prompts for the assessment. The results were recorded by marking the pictures named on a paper recording sheet, computer generated from a picture of the therapy scanning wall (Figure 2). Observations were also recorded to note how the participant carried out the task such as head movements.

Following the baseline assessment, participants had treatment sessions using the scanning wall five days a week for two weeks, with sessions lasting up to 20 minutes. During treatment sessions, encouragement was given to scan through verbal prompts. For example, ‘the tree is in the middle of the wall’ was used to prompt them past midline. If they were unable to see the pictures by scanning initially, they were prompted to identify from one picture to the next. This was to break down the task, so they only needed to scan a short distance, to the next picture, one at a time. Participants were asked questions about pictures on the scanning wall, such as ‘how many birds can you see’? and ‘what colour is the helicopter’? The participants were allowed to move around the room or were assisted in wheel chairs to view the wall from their unaffected sides and interact with the wall to aid learning what the pictures were and where on the wall they were located.

The wall assessment and modified Albert’s test were both repeated 14 days after the baseline assessment.

An acceptability questionnaire was given to all participants on day 14 and also to the treating therapist at the end of the study. The questionnaire aimed to explore perceived benefit, ease of use and duration of the treatment sessions.

## Results

Twenty-two stroke survivors were screened following referral by the therapy team. Twelve were excluded due to cognitive deficits not allowing them to meet the inclusion criteria. A total of 10 stroke survivors with visual inattention were recruited over a 12-month period from a single site. All participants completed the 14-day intervention with no loss to follow up.

The demographics of all recruited participants are outlined in Table 1. Of those recruited, six were female and four were male. The mean age was 69.4 years (SD 14.8). Seven strokes were caused by infarction and three by haemorrhage, all within the right cerebral hemisphere. The mean NIHSS score on admission was 9.3 (SD 4.7), indicating moderate/severe stroke (Kogan et al. 2020).

**Table 1:** Summary of demographics of recruited participants. NIHSS = National Institute of Health Stroke Scale (minimum 0; maximum 42).


PARTICIPANT	GENDER	AGE (YEARS)	TYPE OF STROKE	LATERALITY OF STROKE	ADMISSION NIHSS

1	Male	41	Haemorrhage	Right	19

2	Female	63	Haemorrhage	Right	5

3	Female	96	Infarct	Right	8

4	Male	60	Infarct	Right	16

5	Female	72	Infarct	Right	8

6	Male	67	Infarct	Right	8

7	Female	66	Infarct	Right	10

8	Female	78	Infarct	Right	6

9	Female	84	Infarct	Right	4

10	Male	67	Haemorrhage	Right	9


Table 2 summarises qualitive observations of how the participants identified the pictures at baseline and the final session on day 14. Some participants improved by appearing to learn and remember where pictures were and therefore scanned until they found them.

**Table 2:** Qualitative observations from baseline and day 14 assessments.


PARTICIPANT	INITIAL OBSERVATIONS (BASELINE)	FINAL OBSERVATIONS (DAY 14)

1	Firstly said they could see the tree then scanned from right to left- reported I am a draftsman, so I know to look systematically	Remembered after first session, need to look for pictures- repeated systematic approach so he didn’t miss any.

2	Unable to scan, named pictures on far right only- unaware more pictures	Remembered that they had missed some pictures, a rescanned wall.

3	Unable to scan, named pictures on far right only- unaware more pictures	Remained reduced awareness of deficit, but aware of more pictures on the right only.

4	Sporadically named pictures to midline only – favouring upper quadrant first	More sytematic in approach, once close to midline.

5	Named all pictures in a line down the wall from right to midline to the left, moving head and scanning from one picture to another- missed far left pictures.	Scanned from right to left moving head.

6	Unable to scan, named pictures on far right only- unaware more pictures	Able to scan to midline moving head.

7	Noticed other pictures close by and turned head to scan and find	Remembered that they had missed some pictures, a rescanned wall.

8	Tracked from right to midline only	Freely named all pictures

9	Sporadic naming of pictures	Systematic scanning- But started at the tree and named those to the right before returning to the midline and working from the tree to the left.

10	Noticed more pictures over time and started looking round.	Remained reduced awareness of deficit, but aware of more pictures on the right only.


Initial observation themes were lack of awareness, and therefore unable to scan, and sporadic naming of pictures. Themes for the final observation were that participants strategically scanned from right to left to ensure they did not miss pictures, were aware of the tree as the centre point and demonstrated learning from previous sessions by remembering pictures were there and looking for them. One participant (participant 5) performed better on the scanning wall than on the modified Albert’s test on the initial assessment. During observation, they strategically scanned the wall from right to left and were able to track whilst using the wall. Interestingly, the participant scored full points on both tests by day 14.

The individual scores at both time points, and change over time for both the modified Albert’s tests and scanning wall, are outlined in Table 3. Participants scored a mean of 72.0% (SD 39.2) of the total score for the modified Albert’s test on admission and 96.3% (SD 11.9) on day 14. The scanning wall assessment identified a more severe deficit with a mean of 54.3% (SD 28.1) of the total score on admission and a mean of 85.0% (SD 22.9) on discharge. Both improved by a similar number of percentage points – 24% for the modified Albert’s test and 23% with the scanning wall.

**Table 3:** Individual results for baseline and day 14 assessments (modified Albert’s test and scanning wall) with the difference and percentage change.


PATIENT NUMBER	INITIAL ASSESSMENT		DAY 14 ASSESSMENT		MODIFIED ALBERT’S TEST		SCANNING WALL	
			
	MODIFIED ALBERT’S TEST	SCANNING WALL	MODIFIEDALBERT’S TEST	SCANNING WALL	DIFFERENCE INITIAL TO DAY 14	% CHANGE INITIAL TO DAY 14	DIFFERENCE INITIAL TO DAY 14	% CHANGE INITIAL TO DAY 14

1	40	20	40	30	0	0%	10	34%

2	11	8	40	30	29	73%	22	73%

3	30	6	40	12	10	25%	6	20%

4	40	15	40	26	0	0%	11	37%

5	3	25	40	30	37	93%	5	17%

6	6	3	25	15	19	48%	12	40%

7	40	28	40	30	0	0%	2	7%

8	40	15	40	30	0	0%	15	50%

9	38	23	40	30	2	5%	7	23%

10	40	20	40	22	0	0%	2	7%


Modified Albert’s test (minimum score = 0, maximum score = 40). Scanning wall assessment (minimum score = 0, maximum score = 30).

Nine participants crossed out all lines on the modified Albert’s test on day 14 and six achieved the maximum score with the scanning wall on day 14. All participants showed an improvement using the scanning wall. Participants were included with varying severity of visual inattention, which is demonstrated by a wide range of baseline scores (3–40 modified Albert’s test). All participants demonstrated improvements by day 14.

An exact sign test was used to determine the statistical significance between the modified Albert’s test scores at baseline and at day 14. There was no statistically significant median increase in the modified Albert’s test score (0.5) between baseline (39) and day 14 (40), (*p* = 0.62). A paired-samples t-test was used to determine the statistical significance, the assumption of normality was not violated as assessed by Shapiro-Wilks test (*p* = 0.509). The mean performance on the scanning wall was improved at day 14 to 25.50 (SD 6.88) compared to baseline from 16.30 (SD 8.43). There was a mean improvement of 9.20 (95% CI 4.77 to 13.63) in the 14 days. This is a statistically significant improvement in the scanning wall score between baseline line and day 14 (*p* = 0.01).

On the acceptability questionnaire, 10 participants reported finding the scanning wall beneficial, easy to understand and that the treatment sessions were the right length. This supports the use of the scanning wall as a treatment option for inpatient stroke survivors with visual inattention. Five therapists, either a physiotherapist or an occupational therapist. was involved in the treatment sessions including NS. The remaining four therapists all observed benefits of using the scanning wall as part of the treatment for visual inattention, finding that it was easy to use and could be incorporated into everyday practise with stroke survivors. All answers to the questionnaire were positive.

## Discussion

This proof-of-concept study explored the practical implications and possible benefits of using a scanning wall for acute stroke survivors in the hospital inpatient setting. The scanning wall was able to be used effectively as a treatment alongside standard therapy sessions, and potential benefits were seen.

During assessment, five participants demonstrated no visual inattention using the modified Albert’s test, but a deficit was identified for all participants when using the scanning wall. Correlating with other studies which found that pen and paper tests were not able to detect all visual inattention due to the deficit effecting the extra personal space and wider environment (Azouvi 2017; Van der Stoep et al. 2013). This timely detection of visual inattention affecting the extra personal space during the acute stage post-stroke will in turn lead to earlier intervention, as per the National Stroke Guideline recommendations (Intercollegiate Stroke Working Party 2016). Currently, there is a wide variety of screening assessment for visual inattention, but no gold standard for other assessments to be compared against (Moore et al. 2022; Hanna et al. 2017). The detection of visual inattention by the scanning wall may indicate that it could be a useful screening tool as part of a screening assessment for visual inattention.

After identification, the next step in rehabilitation of visual inattention is facilitating insight into the defect and enabling access to therapeutic strategies (Jehkonen et al. 2000; Longley et al. 2021). The scanning wall has demonstrated that it can support this process. For example, nine participants that initially lacked insight were able to appreciate their deficit when shown using the scanning wall.

Visual scanning training is the theory used to support the scanning wall as therapy for visual inattention. There are numerous visual scanning therapies in the published literature. A recent Cochrane review reported 17 studies that promote stroke survivors to purposefully explore their visual world (Longley et al. 2021). Another strategy used by participants was to start at the far right of the wall and strategically scan across. Five participants could not scan to midline at baseline. In this instance, the participants were moved to show the rest of the wall, then returned to the set standardised position. Three out of the five participants were (immediately from the first session) able to scan further, and by day 14, were able to scan past midline on assessment. Two participants were still only able to scan to midline by day 14, but were improving and therefore may have benefited from further sessions. This may indicate a possible therapeutic effect of the scanning wall.

Results from this proof-of-concept study demonstrated the scanning wall can feasibly be used effectively and safely as an assessment and treatment option within the acute stroke rehabilitation setting. It is an easily administered tool which was low cost to set up. All participants using the scanning wall as a treatment improved from baseline to day 14. The similar change in percentage points between the modified Albert’s test and the scanning wall suggests the scanning wall results are comparable to those of the standardised modified Albert’s test. Initial data show both participants and treating therapists thought the scanning wall was beneficial and easy to use.

There are some limitations to consider for this study. This study delivered the intervention to all participants without a control group to compare the findings against, so it is a possibility that any beneficial effects could have occurred due to standard practice or natural recovery. Full or partial recovery of visual inattention has been reported in 60% of stroke survivors, with the time taken to full recovery being a mean of 54 days (Rowe et al. 2020). All participants within this study were within 54 days post-stroke. The screening for visual inattention in this study used a single pen and paper task, rather than the multiple screening tests which are now recommended (Moore et al. 2022).

## Conclusion

This proof-of-concept study has demonstrated that the scanning wall could be used to assess for visual inattention in extra personal space. Also, the scanning wall could be beneficial and is acceptable for the treatment of visual inattention within a hospital inpatient setting for acute stroke survivors. Further research is required to pilot and assess the feasibility of conducting a randomised controlled trial (RCT) of the scanning wall as a therapeutic option for visual inattention post-stroke. The recommendations of the Cochrane review related to non-pharmacological interventions for inattention following stroke should be followed (Longley et al. 2021). This research should incorporate a wider selection of screening tests to assess the validity of the scanning wall as a screening test.

## References

[1] Azouvi, P. 2017. The ecological assessment of unilateral neglect. Annals of Physical and Rehabilitation Medicine, 60: 186–190. DOI: 10.1016/j.rehab.2015.12.005 26830087

[2] Butler, BC, Lawrence, M, Eskes, GA and Klein, R. 2009. Visual search patterns in neglect: Comparison of peripersonal and extrapersonal space. Neuropsychologia, 47: 869–878. DOI: 10.1016/j.neuropsychologia.2008.12.020 19154749

[3] Checketts, M, Mancuso, M, Fordell, H, Chen, P, Hreha, K, Eskes, GA, Vuilleumier, P, Vail, A and Bowen, A. 2021. Current clinical practice in the screening and diagnosis of spatial neglect post-stroke: Findings from a multidisciplinary international survey. Neuropsychological Rehabilitation, 31: 1495–1526. DOI: 10.1080/09602011.2020.1782946 32691688

[4] Chen, P, Hreha, K, Kong, Y and Barrett, AM. 2015. Impact of spatial neglect on stroke rehabilitation: Evidence fron the setting of an inpatient rehabilitation facility. Archives of Physical Medicine and Rehabilitation, 96: 1458–1466. DOI: 10.1016/j.apmr.2015.03.019 25862254 PMC4519421

[5] Fasotti, L and Van Kessel, M. 2013. Novel insights in the rehabilitation of neglect. Frontiers in Human Neuroscience, 7. DOI: 10.3389/fnhum.2013.00780 PMC3828556 24298249

[6] Halligan, PW, Fink, GR, Marshall, JC and Vallar, G. 2003. Spatial cogntion: Evidence from visual neglect. TRENDS in Cognitive Sciences, 7: 125–133. DOI: 10.1016/S1364-6613(03)00032-9 12639694

[7] Hanna, KL, Hepworth, LR and Rowe, FJ. 2017. Screening methods for post-stroke visual impairment: A systematic review. Disability and Rehabilitation, 39: 2531–2543. DOI: 10.1080/09638288.2016.1231846 27669628

[8] Hepworth, LR, Rowe, FJ, Walker, MF, Rockliffe, J, Noonan, C, Howard, C and Currie, J. 2016. Post-stroke visual impairment: A systematic literature review of types and recovery of visual conditions. Ophthalmology Research, 5. DOI: 10.9734/OR/2016/21767

[9] Intercollegiate Stroke Working Party. 2016. National clinical guideline for stroke. London: Royal College of Physicians. DOI: 10.1034/j.1600-0404.2000.101003195.x

[10] Jehkonen, M, Ahonen, J, Dastidar, P, Koivisto, A, Laippala, P, Vilkki, J and Molnar, G. 2000. Visual neglect as a predictor of function outcome one year after stroke. Acta Neurologica Scandinavica, 101: 195–201. 10705943 10.1034/j.1600-0404.2000.101003195.x

[11] Kogan, E, Twyman, K, Heap, J, Milentijevic, D, Lin, J and Alberts, M. 2020. Assessing stroke severity using electronic health record data: a machine learning approach. BMC Medical Informatics and Decision Making, 20. DOI: 10.1186/s12911-019-1010-x PMC6950922 31914991

[12] Longley, V, Hazelton, C, Heal, C, Pollock, A, Woodward-Nutt, K, Mitchell, C, Pobric, G, Vail, A and Bowen, A. 2021. Non-pharmacological interventions for spatial neglect or inattention following stroke and other non-progressive brain injury. Cochrane Database of Systematic Reviews. DOI: 10.1002/14651858.CD003586.pub4 PMC8247630 34196963

[13] Maxton, C, Dineen, R, Padamsey, R and Munshi, S. 2013. Don’t neglect ‘negelct’–an update on post stroke neglect. International Journal of Clinical Practice, 67: 369–378. DOI: 10.1111/ijcp.12058 23521329

[14] Moore, M, Milosevich, E, Beisteiner, R, Bowen, A, Checketts, M, Demeyere, N, Fordell, H, Godefroy, O, Laczo, J, Rich, T, Williams, L, Woodward-Nutt, K and Husain, M. 2022. Rapid screening for neglect following stroke: A systematic search and European Academy of Neurology recommendations. European Journal of Neurology, 29: 2596–2606. DOI: 10.1111/ene.15381 35510782 PMC9544365

[15] National Institute for for Health and Clinical Excellence. 2013. Stroke rehabilitation in adults. Manchester: National Institute for Health and Clinical Excellence.

[16] Rowe, FJ, Hepworth, LR, Howard, C, Hanna, KL and Currie, J. 2020. Impact of visual impairment following stroke (IVIS study): A prospective clinical profile of central and peripheral visual deficits, eye movement abnormalities and visual perceptual deficits. Disability and Rehabilitation. DOI: 10.1080/09638288.2020.1859631 33347793

[17] Stroke Association. 2022. Stroke statistics [Online]. Available at www.stroke.org.uk/what-is-stroke/stroke-statistics [Last accessed 24 February 2023].

[18] Ten Brink, AF, Biesbroek, JM, Oort, Q and Visser-Meily, JMA. 2019. Peripersonal and extrapersonal visuospatial neglect in different frames of reference: A brain lesion-symptom mapping study. Behavioural Brain Research, 356: 504–515. DOI: 10.1016/j.bbr.2018.06.010 29940260

[19] Van Der Stoep, N, Visser-Meily, JMA, Kappelle, LJ, De Kort, PLM, Huisman, KD, Eijsackers, ALH, Kouwenhoven, M, Van Der Stigchel, S and Nijboer, TC. 2013. Exploring near and far regions of space: Distance-specific visuospatial neglect after stroke. Journal of Clinical and Experimental Neuropsychology, 35: 799–811. DOI: 10.1080/13803395.2013.824555 23984973

[20] Zeltzer, L and Menon, A. 2010. Albert’s Test [Online]. Available at https://strokengine.ca/en/assessments/albert’s-test/ [Last accessed 24 February 2023].

